# Interactions between plant hormones and heavy metals responses

**DOI:** 10.1590/1678-4685-GMB-2016-0087

**Published:** 2017-04-10

**Authors:** Lauro Bücker-Neto, Ana Luiza Sobral Paiva, Ronei Dorneles Machado, Rafael Augusto Arenhart, Marcia Margis-Pinheiro

**Affiliations:** 1 Departamento de Biologia, Universidade Estadual do Centro-Oeste (UNICENTRO), Guarapuava, PR, Brazil; 2 Programa de Pós-Graduação em Genética e Biologia Molecular, Departamento de Genética, Universidade Federal do Rio Grande do Sul (UFRGS), Porto Alegre, RS, Brazil; 3 Empresa Brasileira de Pesquisa Agropecuária - Centro Nacional de Pesquisa de Uva e Vinho, Bento Gonçalves, RS, Brazil

**Keywords:** ABA, auxin, brassinosteroid, ethylene, abiotic stress

## Abstract

Heavy metals are natural non-biodegradable constituents of the Earth's crust that accumulate and persist indefinitely in the ecosystem as a result of human activities. Since the industrial revolution, the concentration of cadmium, arsenic, lead, mercury and zinc, amongst others, have increasingly contaminated soil and water resources, leading to significant yield losses in plants. These issues have become an important concern of scientific interest. Understanding the molecular and physiological responses of plants to heavy metal stress is critical in order to maximize their productivity. Recent research has extended our view of how plant hormones can regulate and integrate growth responses to various environmental cues in order to sustain life. In the present review we discuss current knowledge about the role of the plant growth hormones abscisic acid, auxin, brassinosteroid and ethylene in signaling pathways, defense mechanisms and alleviation of heavy metal toxicity.

## Introduction

During their lifetime, plants are affected by several environmental challenges that threaten survival and negatively influence their growth and productivity. Understanding how plants can translate the signals from an ever-changing environment into physiological behavior is essential for reducing harmful effects caused by abiotic stresses, such as heavy metal toxicity.

Currently, the contamination of natural ecosystems by heavy metals represents a worldwide environmental concern, endangering agricultural systems (Recatala *et al.*, 2006). In developing countries, this problem has arisen from long-term use of untreated wastewater for irrigation, leading to increased concentrations of heavy metals in soils (Arora *et al.*, 2008; Lu *et al.*, 2015). This prevents plants from reaching their maximum genetic potential for growth, development and reproduction.

Once deposited on the ground, plants are able to take up these elements from the soil, and introduce them into the food chain, raising the risk of metal toxicity concerns for humans and animals (Vincevica-Gaile and Klavins, 2012; Roy and McDonald, 2015).

Among heavy metals, some are essential for plant growth and development as micronutrients elements and play an important role in metabolism. Copper and zinc, for example, are critical for the action of Cu- and Zn-dependent enzymes involved in several physiological processes. However, at elevated concentrations, heavy metals negatively affect the morphology, physiology and biochemistry of plants (Gangwar *et al.*, 2010; Zhang *et al.*, 2011; Gautam *et al.*, 2016; Ivanov *et al.*, 2016; Mathur *et al.*, 2016). It has been shown that heavy metals also reduce biomass accumulation (Ghavri and Singh, 2012; Zhao *et al.*, 2012; Beyer *et al.*, 2013; Ebbs *et al.*, 2015) as a result of adverse effects upon key metabolic processes such as photosynthesis (Rodriguez *et al.*, 2012), mineral nutrition (Vernay *et al.*, 2007) and interactions with water (Mukhopadhyay and Mondal, 2015).

Besides the direct impact of heavy metals on plants, they can also cause cell toxicity by overproduction of reactive oxygen species (ROS), which impairs antioxidant defense systems and causes oxidative stress (Vanhoudt *et al.*, 2010; Thounaojam *et al.*, 2012; Anjum *et al.*, 2016; Nanda and Agrawal, 2016; Rui *et al.*, 2016).

In plant cells, aerobic reactions such as respiration or photosynthesis lead to ROS production. These ROS, such as hydroxyl, hydrogen peroxide or superoxide, can damage biological molecules, including lipids, DNA and proteins (Noctor and Foyer, 1998). Rates of ROS generation and cellular ROS levels both increase quickly when plants are subjected to abiotic or biotic stress.

Bioactive-metals, based on their physicochemical properties, are divided into two groups: redox metals such as Cr, Cu, Mn, and Fe, and non-redox metals such as Cd, Ni, Hg, Zn, and Al. The redox metals can directly generate oxidative injury in plants through Haber-Weiss and Fenton reactions, which leads to production of ROS, resulting in oxidative stress via disequilibrium between prooxidant and antioxidant homeostasis (Jozefczak *et al.*, 2012). In contrast, non-redox metals indirectly act as oxidative stressors by several mechanisms including glutathione depletion, binding to sulfhydryl groups of proteins, inhibiting antioxidative enzymes or inducing ROS-producing enzymes like NADPH oxidases (Bielen *et al.*, 2013). Thus, high concentrations of contaminants affect plants from molecular to physiological levels. However, the precise mechanisms involved in these processes are not well understood.

Previous studies have shown that exogenous application of plant growth hormones (also known as plant hormones or phytohormones) can improve protection against heavy metal toxicity (Al-Hakimi 2007; El-Monem *et al.*, 2009; Zhu *et al.*, 2012; Agami and Mohamed 2013; Zhu *et al.*, 2013; Masood *et al.*, 2016). Acting as chemical messengers with highly complex regulation, these molecules allow plants to retain growth plasticity during development and collectively are probably the main means by which plants respond to abiotic and biotic stresses (Nishiyama *et al.*, 2011; Chan 2012; Colebrook *et al.*, 2014; Xu *et al.*, 2016).

In plants, the chelation and sequestration of heavy metals by the best-characterized ligands phytochelatin and metallothionein is an important mechanism in the uptake and accumulation of both essential and nonessential heavy metals. These metal-binding cysteine-rich proteins hammer out complexes that accumulate in the vacuole and ultimately alleviate metal toxicity (Clemens, 2001; Clemens and Ma, 2016). There is also some evidence that phytohormones can be involved in phytochelatin biosynthesis. In the algae *Chlorella vulgaris*, brassinosteriods increased the total phytochelatin content under lead stress (Bajguz, 2002). In the same way, it has been suggested that there is a participation of abscisic acid in the regulation of phytochelatin synthase in potato tubers (Stroinski *et al.*, 2010). Although possible, a clear link between heavy metals, hormonal pathways and metal-binding ligands in plants still needs to be explained.

In view of the significance of several phytohormones such as gibberellic acid and cytokinin as crucial key players in metal stress mitigation, (El-Monem, 2009; Zhu *et al.*, 2012; Masood *et al.*, 2016; Al-Hakimi 2007; Gangwar *et al.*, 2010), this review discusses recent discoveries related to heavy metal contamination and the role of three well-studied plant hormones (abscisic acid, auxin and ethylene). Also, we present the new findings related to the plant steroid hormone brassinosteroid, taking into account their potential significance in signaling, defense mechanisms and alleviation of the toxic effects of heavy metal exposure.

## Abscisic acid

Abscisic acid (ABA) is a multifunctional phytohormone that plays an important role during many stages of a plant's life cycle, including seed development and dormancy (Nambara *et al.*, 2010; Finkelstein 2013). This hormone has been linked with tolerance to adverse environmental conditions, and its signaling pathway is a central regulator of abiotic stress response in plants (Bartels and Sunkar, 2005; Tuteja, 2007; Danquah *et al.*, 2014).

ABA concentration in plant tissues is known to increase after heavy metal exposure, suggesting an involvement of this phytohormone in the induction of protective mechanisms against heavy metal toxicity (Rauser and Dumbroff, 1981; Poschenrieder *et al.*, 1989; Hollenbach *et al.*, 1997).

It was shown that cadmium (Cd) treatment leads to increased endogenous ABA levels in roots of *Typha latifolia* and *Phragmites australis* (Fediuc *et al.*, 2005), in potato tubers (Stroinski *et al.*, 2010) and also in rice plants (Kim *et al.*, 2014). The same effect was verified in several other studies. When mercury (Hg), Cd and copper (Cu) solutions were applied to wheat seeds during germination, ABA levels increased (Munzuro *et al.*, 2008). In cucumbers, seed germination decreased and ABA content increased under Cu^2+^ and zinc (Zn^2+^) stress (Wang *et al.*, 2014c). Similarly, augmented amounts of ABA were detected in germinating chickpea (*Cicer arietinum*) seeds under lead (Pb) toxicity conditions (Atici *et al.*, 2005), as well as in crowberries (*Empetrum nigrum*) exposed to Cu and nickel (Ni) (Monni *et al.*, 2001).

Transcriptome analysis of a rice under arsenic (As) stress has revealed strong expression of ABA biosynthesis genes *OsNCED2* and *OsNCED3*, as well as the up-regulation of four ABA signaling genes under metal stress (Huang *et al.*, 2012). In a pioneering approach, Lin *et al*. (2013) used a whole-genome array to perform a transcriptomic analysis of rice roots exposed to vanadium (V) and showed that this metal triggered the expression of genes associated with signaling and biosynthesis of ABA.

The complex regulation of ABA is mediated by three main components of its signaling pathway, the PYL/PYR/RCAR, PP2C and SnRK2. In an attempt to clarify the transcriptional regulation of ABA signal transduction during cucumber seed germination under Cu^2+^ and Zn^2+^ exposure, Wang *et al*. (2014c) identified nine *PYL*, three *PP2C* and two *SnRK2* genes that are putatively involved in ABA signal transduction. The expression patterns of these genes were also investigated, and the results showed that gene expression varied with the treatment being used. However, further studies are necessary in order to better understand how core component genes of ABA signal transduction mediate heavy metal stress through gene expression induction.

Since the ABA concentration increases in response to heavy metal stress, its potential role as a mediator of Cd-induced phytotoxicity was examined (Sharma and Kumar, 2002). Results obtained from ABA-deficient and ABA-insensitive mutants exposed to Cd ruled out the involvement of this phytohormone in coordinating Cd-imposed inhibitory effects on early growth.

Despite a lack of knowledge regarding the way in which the ABA signaling pathway changes in response to heavy metal exposure, a series of results indicate a strong correlation between high levels of ABA and a decrease in plant stress. Furthermore, some evidence has shed light on the functional significance of elevated hormone concentrations in plants exposed to heavy metal toxicity.

ABA is normally considered a signaling compound, which can be extracted from roots subjected to drought conditions. Transported via xylem sap to the guard cells, where it closes stomata, this phytohormone prevents a decline in water potential and contributes to the plant's adaptation to unfavorable conditions (Sauter *et al.*, 2001; Wilkinson and Davies 2002; Pantin *et al.*, 2013). It is well established that exposure to toxic metal concentrations impairs plant water balance (Rauser and Dumbroff, 1981; Schat *et al.*, 1997; Mukhopadhyay and Mondal, 2015). Therefore, ABA, in this context, could play an important role in the protection against abiotic stress.

Nickel and zinc treatments have been shown to decrease water potential and stomatal conductance of 10 day-old white bean seedlings, whilst ABA levels increased (Rauser and Dumbroff, 1981). A similar finding was reported in *Brassica juncea* under Cd^2+^ exposure (Salt *et al.*, 1995). Cd-treated white bean seedlings showed decreased relative water uptake rate whilst stomatal resistance and ABA content increased (Poschenrieder *et al.*, 1989). It has previously been suggested that drought resistance could help plant survival in contaminated areas (Pandolfini *et al.*, 1996).

It is possible that ABA-induced stomatal closure causes a suppression of transpirational flow, resulting in a restriction of root-to-shoot translocation of metals. Previously it was reported that Cd (Yeh *et al.*, 2003) and Cu (Yeh *et al.*, 2004) treatments boost mitogen-activated protein kinase (MAPK) signaling in rice, and that an increase in ABA content in specific rice varieties is closely related to Cd tolerance in seedlings (Hsu and Kao, 2003). More recently, Yeh *et al*. (2007) showed higher levels of ROS and calcium (Ca^2+^) in the same Cd-tolerant rice seedlings. These results indicate that roots under heavy metal exposure accumulate ROS and Ca^2+^, which in turn activate MAP kinase, providing Cd tolerance. It is already known that ABA is able to induce transient MAP kinase activity (Knetsch *et al.*, 1996; Burnett *et al.*, 2000), and that ABA signaling seems not to be required for ROS production, suggesting that ROS may be upstream of ABA biosynthesis (Galvez-Valdivieso *et al.*, 2009). Despite the progress achieved, more work is required in order to unravel the role of ABA in MAP kinase activation in response to heavy metal toxicity.

Rubio *et al*. (1994) showed that exogenous ABA applications affected the transport of Cd and Ni to the shoots, resulting in a higher percentage of metals in the root. In *Arabidopsis thaliana*, the exogenous hormone limited the root-to-shoot translocation of Cd (Perfus-Barbeoch *et al.*, 2002). ABA restriction of heavy metal transport to the shoots may be of practical importance, at least for plants growing in Ni-polluted soils, since it is known that this metal can accumulate in fruits and seeds due to its high mobility in the phloem (Rubio *et al.*, 1994).

There is also some evidence that ABA's potential role in plant response to heavy metal stress is related to growth inhibition. Moya *et al*. (1995) showed that rice cultures supplied with ABA displayed enhanced heavy metal toxicity, causing growth inhibition of young leaves and the translocation of storage products from source to sink organs. This finding is in accordance with previous observations of phloem-loading inhibition by ABA (Vreugdenhil, 1983), which could account for the accumulation of assimilates in the source and subsequent inhibition of plant growth. This process may be an adaptation to maintain viability under adverse conditions, and further enable recovery once the toxin is eliminated from the environment.

**Figure 1:**
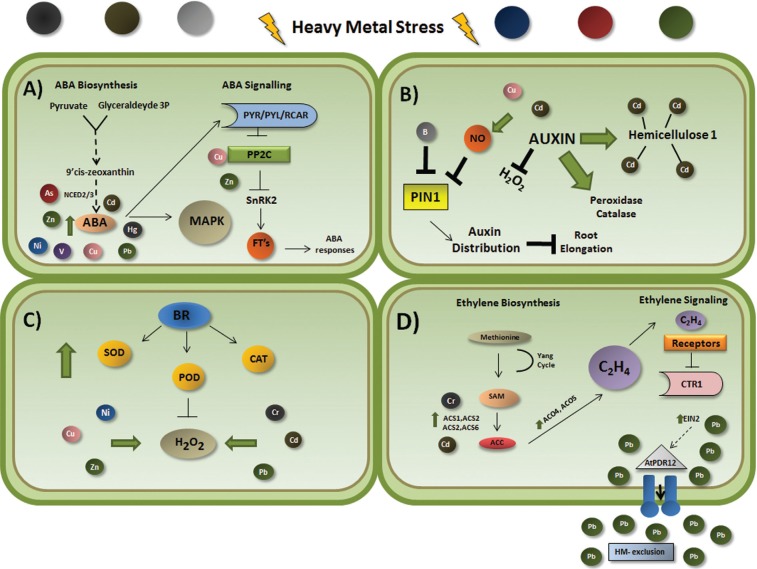
Schematic representation showing some interactions between the plant hormones abscisic acid (A), auxin (B), brassinosteroids (C) and ethylene (D) under heavy metal exposure. (A) Cd, Cu, Hg, Ni, Pb and Zn treatments increase endogenous levels of ABA. Under As stress, *NCED2* and *NCED3* (ABA biosynthesis genes) are up-regulated. Vanadium (V) is also shown to trigger the expression of genes associated with ABA signaling and biosynthesis. The genes *PYL*, *PP2C* and *SnRK2* that are putatively involved in ABA signal transduction were identified under Cu and Zn exposure. (B) Under B starvation PIN1 changes auxin distribution and possibly inhibits root elongation. Cd induces NO accumulation, which represses auxin transport and reduces root meristem size. NO is also involved in the auxin signaling pathway in response to Cu exposure. Under Cd stress, an auxin conjugate (IAA-Asp) modulates catalase and peroxidase activity and decreases hydrogen peroxide concentration. In the same condition, auxin (NAA) increases hemicellulose 1 content and more Cd is fixed in the roots. (C) BRs induce SOD, CAT and POD activities, protecting plants against heavy metal toxicity. (D) *ACS* and *ACO* expression leads to higher production of ethylene. *ACS2* and *ACS6* are regulated by MPK3/MPK6 at transcriptional and translational levels. MPK3 and MPK6 phosphorylate the transcription factor WRKY33, which in turn regulates *ACS2* and *ACS6* gene expression. The WRKY33 protein also binds directly to *ACS2* and *ACS6* promoters. *EIN2* is an important component of the ethylene signaling pathway and acts as a transducer of stress response. Lead treatment increased the transcript levels of *EIN2* in Arabidopsis seedlings under heavy metal exposure. It has been suggested that EIN2 regulates AtPDR12, an ABC membrane-transporter that excludes Pb and Pb-containing toxic compounds from the cytoplasm. Dashed black line indicates possible regulation. Arrows and T-bars represent positive and negative regulation, respectively. Green arrows indicate increased levels. As, arsenic; B, boron; Cd, cadmium; Cr, chromium; Cu, copper; Hg, mercury; Ni, nickel; Pb, lead; Zn, zinc; V, vanadium; FT's, transcription factors; NO, nitric oxide; BRs, brassinosteroids; SOD, superoxide; CAT, catalase; POD, peroxidase; ACS, ACC synthetase; ACO, ACC oxidase.

In combination, these studies suggest that ABA may be a crucial player in plant response to heavy metal toxicity. However, further studies are required in order to determine how this plant growth hormone guides adaptation under challenging conditions. Some interactions between ABA and heavy metals are shown in Figure 1A.

## Auxin

The plasticity of plant development and its response to a diversity of environmental cues including the fine-tuning of stress management indicates that regulatory processes are very complex. Among these environmental situations faced by plants, the interaction between auxin homeostasis and heavy metal toxicity is of particular interest, once this phytohormone (representative molecule - indole-3-acetic acid, IAA) is often reported as an important mediator in several aspects of plant growth and development.

Inside the cell, the hormone promotes the degradation of AUX/IAA transcriptional repressors that are associated to ARFs (auxin responsive factors) and thus inhibit the transcription of auxin-responsive genes ultimately guiding modifications in plant physiology (Vanneste and Friml, 2009).

Auxin metabolism and dynamic polar transport distribution within the plant can be modulated by heavy metal stimuli. Results obtained from a range of heavy metal concentrations in DR5:GUS reporter line indicated that hormone accumulation and patterning in Arabidopsis seedling is treatment-dependent (Wang *et al.*, 2014a), and an important role has been credited to the PINFORMED1 (PIN1) protein, an auxin efflux carrier for root meristem growth/maintenance under normal conditions. According to Yuan *et al*. (2013), when compared to DR5rev::GFP plants, Cu-treated *pin1* DR5rev::GFP mutants did not show enhanced auxin activity in both the meristem and elongation zones, indicating that PIN1 is involved in hormone distribution under heavy metal stress conditions. In a similar way, auxin distribution is also altered under boron (B) starvation, which leads to PIN1 down-regulation and possible inhibition of root elongation (Li *et al.*, 2015). This effect in root elongation could be an indirect effect caused by the crosstalk between auxin and other hormones, such as ethylene. As reported by Ruzicka et *al.*, (2007), ethylene induces the expression of genes involved in auxin biosynthesis and stimulates the auxin transport toward the elongation zone, regulating its response and consequently root elongation. In Arabidopsis plants exposed to boron deficiency, auxin, ethylene and ROS participate together in a signaling pathway, acting in the reduction of root cell elongation (Camacho-Cristóbal et *al.*, 2015). In addition, there are many examples, explored later in this review, showing that heavy metal toxicity can induce ethylene accumulation, which suggests a possible relation between auxin and ethylene signaling under heavy metal stress.

The roles of nitric oxide (NO) and auxin in Cd-mediated inhibition of Arabidopsis root meristem growth has also been investigated (Yuan and Huang, 2016) and the results indicate that Cd induced NO accumulation, which in turn repress auxin transport, decreasing its levels in the root apex and ultimately reducing root meristem size. It is interesting to note that NO was previously shown to be involved in the auxin signaling pathway under Cu stress conditions (Peto *et al.*, 2011).

Generally, heavy metal stress leads to a decrease in endogenous levels of auxins. For example, arsenic (As) is able to alter levels of three auxins (IAA, NAA, and indole-3-butyric acid, IBA) in *Brassica juncea* (Srivastava *et al.*, 2013). In another case, short-term cadmium treatment disturbed IAA homeostasis in barley root tips (Zelinová *et al.*, 2015). Previous work also indicates that Cadmium (Cd) suppresses primary root elongation in Arabidopsis (Besson-Bard *et al.*, 2009).

Despite the detrimental effect of heavy metal in auxin metabolism, it has been reported that exogenous application of these phytohormones can rescue the endogenous levels of the auxins. An increase in the biomass of roots and stems of sunflower (*Helianthus annuus*) plants grown in soil moderately contaminated with lead (Pb) was observed after the addition of the phytohormone IAA (Liphadzi *et al.*, 2006). Exogenous supply of IAA also improved the growth of *Brassica juncea* exposed to As (Srivastava *et al.*, 2013). In the same way, the application of different levels of L-TRP (a precursor of auxin) to the roots of rice seedlings growing in contaminated soil enhanced plant growth and yield under Cd stress, when compared to untreated seedlings in Cd-contaminated pots without this auxin precursor (Farooq *et al.*, 2015).

Some recent approaches showed that this synergistic or additive interaction between heavy metal and auxin can be used as a protective mechanism against toxicity in crop plants or as a useful tool in phytoremediation programs for detoxification of polluted areas. Tandon *et al*. (2015) evaluated the application of six concentrations of two representative natural auxins (IAA and IBA), and a synthetic auxin (1-Naphathaleneacetic acid), in wetland and non-wetland plant species in a water environment. The authors showed that exogenous auxin supply increased phytoremediation efficiency in wastewater treatment. Similarly, Pandey and Gupta (2015) studied the effect of the co-application of selenium (Se) and auxin on morphological and biochemical characteristics in rice seedlings exposed to As stress. When used together, Se and auxin were more effective in reducing As-induced stress compared to individual treatments.

Although the exogenous addition of auxin or stimulation of endogenous levels prevents growth inhibition and increase heavy metal tolerance, the mechanism guiding the process is still poorly understood. It is possible that there is a relationship between hormones and miRNAs under heavy metal exposure (Srivastava *et al.*, 2013).

In another study, Ostrowski *et al*. (2016) showed that IAA-Asp (an auxin conjugate) affects pea responses to Cd by modulating catalase and peroxidase activity, as well as inducing protein carbonylation and decreasing hydrogen peroxide concentration.

There is also evidence indicating that auxins can promote modification of membrane properties, thereby alleviating toxic effects of heavy metal exposure. A mixture containing lead (Pb^2+^) and IAA or naphthalene acetic acid (NAA - another member of the auxin family) has been proposed to induce decreased disorder of membrane organization, and as a consequence, reduce heavy metal toxicity (Hac-Wydro *et al.*, 2016).

It has also been suggested that hemicellulose 1 in the cell wall is a key player in heavy metal detoxification. Under Cd^2+^ exposure, NAA increases metal retention in roots by fixing it to hemicellulose (Zhu *et al.*, 2013). Exogenous NAA enhances hemicellulose 1 content and consequently the amount of Cd^2+^ fixed in the roots. In this way, auxin-induced alleviation of Cd^2+^ toxicity in Arabidopsis is mediated by increased levels of hemicellulose 1 and metal fixation in the root, thus reducing the translocation of Cd^2+^ from roots to shoots.

Apart from deleterious effects in auxin homeostasis, it is interesting to note that the interaction between heavy metals and the phytohormone seems to be critical for survival and reproduction of some organisms. It was recently reported that the metallophyte (tolerant to high concentrations of heavy metals) moss *Scopelophila cataractae* requires a Cu-rich environment to maintain its life cycle. Under this “favorable” condition, auxin accumulates and in turn activates genes required for optimal growth and cell differentiation, although the precise mechanism is not yet elucidated (Nomura *et al.*, 2015).

These studies indicate a complex regulation of endogenous auxin in response to heavy metal exposure (Figure 1B). It is possible to speculate that several crosstalk may act concomitantly in a not-well understood signaling pathway and that a better comprehension of these processes is critical to increase the knowledge on the regulation of metal homeostasis in plants.

## Brassinosteroids

Plant steroids (brassinosteroids – BRs) regulate cell expansion and elongation, photomorphogenesis, flowering, male fertility, seed germination, vascular differentiation, plant architecture, stomata formation and senescence in plants (Mandava, 1988). Sixty BRs have been identified so far and are classified according to the number of carbons in their structure (Vardhini, 2014). Brassinolide (BL), 28-homobrassinolide (28-HomoBL) and 24-epibrassinolide (24-EpiBL) are the bioactive BRs widely used in studies (Vardhini *et al.*, 2006).

In addition to their significance in general plant growth and development, BRs perform a variety of physiological roles in guarding against abiotic stress, including high and low temperatures (Janeczko *et al.*, 2011; Wang *et al.*, 2014b), salinity (Abbas *et al.*, 2013), light (Wang *et al.*, 2010), drought (Mahesh *et al.*, 2013), and herbicides and pesticides (Xia *et al.*, 2006; Sharma *et al.*, 2013).

One of the main reactions of plants when subjected to stress is the elevated generation of ROS. These include the radicals superoxide (O_2_^-^), hydroxyl (OH^-^), perhydroxyl (HO_2_^-^), alkoxy (RO), as well as the non-radicals hydrogen peroxide (H_2_O_2)_ and singlet oxygen (O_2_) (Anjum *et al.*, 2010; Gill and Tuteja 2010; Anjum *et al.*, 2012; Anjum *et al.*, 2015). Plant stress tolerance requires the activation of complex metabolic activities including antioxidant pathways (ROS-scavenging system) within cells, that in turn contribute to plant growth under stress conditions (El-mashad and Mohamed 2012). Plant antioxidant defense systems consist of enzymes such as superoxide (SOD), catalase (CAT), peroxidase (POD), ascorbate peroxidase (APX), glutathione reductase (GR) and glutathione sulfotransferase (GST). Excess of ROS and their reaction products that escape antioxidant-mediated scavenging systems cause oxidative stress, leading to critical damage to the primary metabolites of plants (Anjum *et al.*, 2010, 2012, 2015; Gill and Tuteja, 2010). It is well known that heavy metal phytotoxicity is deeply related to oxidative stress and the consequent production of ROS in plants. Interestingly, antioxidant enzyme activities are also regulated by BRs (Cao *et al.*, 2005). Indeed, BR-induced stress tolerance is associated with an increased expression of genes with antioxidant functions (Xia *et al.*, 2009).

The heavy metal nickel (Ni) is another important environmental contaminant. High concentrations of Ni^2+^ ions can bind to proteins and lipids inducing oxidative damage (Bal and Kasprzak, 2002). Exogenous application of 24-epiBL has been found to ameliorate Ni-stress in *Brassica juncea* by enhancing the activity of antioxidant enzymes (Kanwar *et al.*, 2013). In the same way, elevated CAT, POD, and SOD activity by exogenous application of 28-homoBL protects wheat against Ni toxicity (Yusuf *et al.*, 2010). Elevated antioxidant activity in response to Ni was also found in *Raphanus sativus* and *Vigna radiate* pre-treated with 24-epiBL (Sharma *et al.*, 2011a; Yusuf *et al.*, 2012).

Cadmium (Cd) is known to be toxic even at a very low concentrations as it accumulates in edible parts of growing plants, thereby endangering crop yield and quality (Vázquez *et al.*, 2013). Foliar application of homoBL improves Cd-tolerance in *Brassica juncea* by increasing activity of antioxidant enzymes (CAT, POD and SOD) (Hayat *et al.*, 2007). Using *Phaseolus vulgaris* as a model, enhanced Cd tolerance was possible with exogenous application of 24-epiBL (Rady, 2011). In tomatoes, reduced damage by Cd was reported with application of 28-homoBL/24-epiBL (Hasan *et al.*, 2011). The exogenous application of BRs in Cd-stressed *Solanum lycopersicum* plants enhanced antioxidant system activity and improved fruit yield and quality (Hayat, 2012). A similar effect was reported using 28-homoBL in the protection of *Cicer arietinum* against Cd (Hasan *et al.*, 2008).

Copper (Cu) is an essential transition metal, and an indispensable component in a diverse range of plant metabolic reactions. Furthermore, Cu has become increasingly hazardous due to its inclusion in fungicides, fertilizers and pesticides (Sonmez *et al.*, 2006). BR treatment mitigates the effect of excess Cu by reducing H_2_O_2_ content and increasing CAT, POD, and SOD activities in *Brassica juncea* and *Raphanus sativus* plants (Fariduddin *et al.*, 2009; Kapoor *et al.*, 2014). Supplementation of BRs also helps plants to enhance antioxidant enzymatic activities in response to other heavy metals such as zinc (Arora and Bhardwaj, 2010; Ramakrishna and Rao, 2013), lead (Anuradha and Rao, 2007; Rady and Osman, 2012) and chromium (Choudhary *et al.*, 2011; Sharma *et al.*, 2011b).

These results all suggest that BRs play a crucial role in response to heavy metal toxicity (Figure 1C). Their ability to improve the effectiveness of antioxidant systems by elevating the activity and levels of enzymatic and non-enzymatic antioxidants has made their use a potential strategy for increasing crop resistance to heavy metal stress.

## Ethylene

The plant growth hormone ethylene is involved in many developmental processes, such as the “triple response” in seedlings (leaf abscission, fruit ripening and senescence). Several studies have reported the involvement of this phytohormone in response to abiotic stress, and previous experiments have shown an increased production of ethylene in plants exposed to toxic levels of heavy metals (Maksymiec, 2007; DalCorso *et al.*, 2010; Khan *et al.*, 2015). However, the way in which ethylene regulates mechanisms of heavy metal tolerance remains unclear.

Ethylene is synthesized from methionine, which is converted to S-adenosylmethionine (SAM) by SAM synthetase. SAM, as a substrate, is capable of forming 1-aminocyclopropane-1-carboxylic acid (ACC) by ACC synthase (ACS). This is the rate-limiting step in the ethylene biosynthesis pathway and ultimately releases 5′-methylthioadenosine (MTA), which is recycled again to methionine via the so-called “Yang cycle.” However, in the presence of O_2_, ACC is degraded by ACC oxidase (ACO) to form ethylene, CO_2_ and cyanide in plants (Yang and Hoffman, 1984). The enzymes ACS and ACO are the two fundamental components of ethylene biosynthesis. Heavy metal stress increases the expression of genes encoding these two proteins, resulting in higher phytohormone production (Schellingen *et al.*, 2014; Khan *et al.*, 2015).

Copper (Cu) has been found to induce the expression of the ACS genes in potatoes, and in the same way, different varieties of tobacco accumulated ACS transcripts under Cu exposure (Schlagnhaufer *et al.*, 1997).

Transcriptome analysis of chromium (Cr)-treated rice roots showed increased expression of four ethylene biosynthesis-related genes (ACS1, ACS2, ACO4, and ACO5), indicating the participation of ethylene in Cr signaling in rice (Steffens, 2014; Trinh *et al.*, 2014). Cadmium (Cd) induced the biosynthesis of ACC and ethylene in *Arabidopsis thaliana* via the increased expression of ACS2 and ACS6 (Schellingen *et al.*, 2014). These results were confirmed by observations in *acs2-1acs6-1* double knockout mutants, which showed a decrease in ethylene production. The reduced amount of phytohormone affected leaf biomass and resulted in a delayed induction of ethylene responsive genes without significant differences in Cd content between wild-type and mutant plants, indicating that the decrease in ethylene production in mutants is not related to a lower Cd uptake (Figure 1D).

Together, these findings demonstrate that under heavy metal stress conditions, plants show a rapid increase in ethylene production and reduced plant growth and development, indicating a strong involvement of this phytohormone in plant response to heavy metal toxicity (Maksymiec, 2007; Schellingen *et al.*, 2014).

Under heavy metal exposure, the enzymes of ethylene biosynthesis, ACS2 and ACS6, are phosphorylated by MAPKs, which in turn increase their half-life. Both phosphorylated and native ACS forms are functional, however the former is more stable and active compared to the latter (Liu and Zhang 2004; Joo *et al.*, 2008; Skottke *et al.*, 2011). Moreover, MPK3 and MPK6 are able to phosphorylate the transcription factor WRKY33, which in turn regulates *ACS2* and *ACS6* gene expression. The WRKY33 protein binds directly to the W-boxes in the promoters of *ACS2* and *ACS6* genes *in vivo*, suggesting that WRKY33 also activates *ACS2* and *ACS6* expression downstream of the MPK3/MPK6 cascade (Li *et al.*, 2012). It is important to emphasize that most studies have focused on *ACS* or *ACO* gene expression levels, although other levels of regulation, such as post-transcriptional and post-translational modifications, can affect the abundance of enzyme activity under heavy metal exposure (Figure 1D).

The Arabidopsis *EIN2* gene is an important component of the ethylene signaling pathway and acts as a transducer of ethylene and stress responses (Alonso *et al.*, 1999). Cao *et al*. (2009) showed that lead (Pb) treatment increased transcript levels of *EIN2* in Arabidopsis seedlings, indicating a putative role of the gene in heavy metal tolerance (Figure 1D). They also reported that in several developmental stages, there was no difference between an *ein2-1 mutant* and wild-type seedlings. However, when grown on culture medium containing different concentrations of Pb, the *ein2-1* mutants were more sensitive to the metal than wild-type seedlings (Cao *et al.*, 2009).

The efflux pump at the plasma membrane is one of the most important mechanisms contributing to Pb resistance in Arabidopsis, and the *AtPDR12* is an ABC membrane-transporter gene that excludes Pb and Pb-containing compounds from the cytoplasm (Lee *et al.*, 2005). In *ein2-1* mutant seedlings the transcript levels of *AtPDR12* were lower than in wild-type plants either in the absence or presence of Pb (Lee *et al.*, 2005). This result leads to infer that the *EIN2* gene may be involved in the regulation of the *AtPDR12* gene, and that the increased sensitivity of *ein2-1* mutant seedlings to Pb is, at least partially, correlated with reduced expression of the *AtPDR12* gene. However, it is still not known how the *EIN2* gene mediates the expression of *AtPDR12* (Cao *et al.*, 2009) (Figure 1D).

There are also some interesting results showing that Arabidopsis ethylene-insensitive *etr1-1* and *ein3-3* mutants are less sensitive to lithium than wild type plants (Bueso *et al.*, 2007). The genes *ETR1* and *EIN3* act in the ethylene signaling pathway as a receptor and a transcription factor, respectively (Wang *et al.*, 2002). This controversial result may be attributed to metal-specific properties, or differences in the experimental conditions such as metal concentration, exposure time, plant species and developmental stage.

Despite progress made in recent years, more work is required in order to gain a better understanding of ethylene biology and its relationship with heavy metal stress tolerance in a molecular and physiological context. This will increase our knowledge of the molecular mechanisms of plant response to heavy metal toxicity.

## Future Perspectives

Increasing efficiency in the use of resources, improving the quality of agricultural products, and the preservation of natural resources are key challenges facing modern agriculture. Environmental stresses, either natural or anthropogenic, can significantly reduce the potential of crop yields and restrict the sites and soils where commercially important species can be grown. Abiotic stresses such as drought, excessive rainfall, very high or low temperatures, low light, and heavy metal toxicity, are a constant threat to the development of agricultural practices.

The increase of heavy metals in various terrestrial and aquatic ecosystems has led to concerns relating to the spread of these elements in concentrations that could compromise the quality of the environment. Recently, much attention has been given to the development of strategies designed to alleviate the adverse effects of heavy metal toxicity on crops, and the large amount of research in this area has significantly expanded our understanding of molecular mechanisms involved in metal uptake (Kanwar *et al.*, 2013; Roy and McDonald, 2015), transport (Rubio *et al.*, 1994; Lin *et al.*, 2013) and detoxification (Lee *et al.*, 2005; Zhu *et al.*, 2013; Tandon *et al.*, 2015) in plants (Table 1). However, several key components of the complex metal-signaling network in plants are still to be elucidated.

**Table 1:** Interrelation between phytohormone and heavy metal treatments.

Hormones	Treatment	Effect	Plant	References
**ABSCISIC ACID**	Cd	Increased endogenous ABA levels	*Typha latifolia* and *Phragmites australis*	Fediuc *et al*. (2005)
			Potato tubers	Stroiñski *et al*. (2010)
			Rice plants	Kim *et al*.
				
	Hg, Cd and Cu	Increased ABA levels	Wheat seeds	Munzuro *et al*. (2008)
	Cu and Zn	Seed germination decreased and ABA	Cucumbers	Wang *et al*. (2014c)
		content increased		
	Pb	ABA accumulation	*Cicer arietinum*	Atici *et al*. (2005)
				
	Cu and Ni	ABA accumulation	*Empetrum nigrum*	Monni *et al*. (2001)
	As	Induction of ABA biosynthesis and signaling genes	Rice	Huang *et al*. (2012)
	V	Induction of ABA biosynthesis and signaling genes	Rice	Lin *et al*. (2013)
	Cu and Zn	Induction of genes related to ABA signal transduction	Cucumber	Wang *et al*. (2014)
	Cd	Inhibitory effects on early growth	*Arabidopsis thaliana*	Sharma and Kumar (2002)
	Ni and Zn	*Decreased water potential and stomatal conductance and increased ABA levels*	White bean	Rauser and Dumbroff (1981)
	Cd	Decreased water potential and stomatal conductance and increased ABA levels	*Brassica juncea*	Salt *et al*., 1995
	Cd	Decreased relative water uptake rate whilst stomatal resistance and ABA content increased	White bean	Poschenrieder *et al*., 1989
	Cd and Cu	Induced MAPK signaling and increased ABA content	Rice	Yeh *et al*. (2003, 2004)
	Exogenous ABA application	Affected the transport of Cd and Ni to the shoots	Rice	Rubio *et al*. (1994)
		Limited the root-to-shoot translocation of Cd	*Arabidopsis thaliana*	Perfus-Barbeoch *et al*. (2002)
		Enhanced heavy metal toxicity, causing growth inhibition and the translocation of storage products from source to sink organs	Rice	Moya *et al*. (1995)
**AUXIN**	Cu	Enhanced auxin activity in both the meristem and elongation zones	*Arabidopsis thaliana*	Yuan *et al*. (2013)
	B	PIN1 down-regulation and inhibition of root elongation.	*Arabidopsis thaliana*	Li *et al*. (2015)
	Cd	Induced NO accumulation, repress auxin transport and reduced root meristem size	*Arabidopsis thaliana*	Yuan and Huang, (2016)
	Cu	Increased auxin and decreased NO levels in roots	*Arabidopsis thaliana*	Peto *et al*. (2011)
	As	Changed IAA, NAA and IBA levels	*Brassica juncea*	Srivastava *et al*. (2013)
	Cd	Disturbed IAA homeostasis.	Barley	Zelinová *et al*. (2015)
		Suppressed primary root elongation	Arabidopsis	Besson-Bard *et al*. (2009)
	Exogenous IAA application	Increased the biomass of roots in soil moderately contaminated with Pb	*Helianthus annuus*	Liphadzi *et al*. (2006)
		Improved growth after exposition to As	*Brassica juncea*	Srivastava *et al*. (2013)
	Exogenous of auxin precursor	Enhanced plant growth and yield under Cd stress	Rice	Farooq *et al*. (2015)
	Exogenous natural and synthetic auxin application	Increased phytoremediation efficiency in wastewater treatment	Wetland and non-wetland	Tandon *et al*. (2015)
	Co-application of selenium (Se) and auxin	Reduced As-induced stress	Rice	Pandey and Gupta (2015)
	IAA-Asp	Modulated catalase and peroxidase activity, induced protein carbonylation and decreased hydrogen peroxide concentration	Pea	Ostrowski *et al*. (2016)
	Exogenous NAA application	Enhanced hemicellulose 1 content and the amount of Cd^2+^ fixed in the roots	Arabidopsis	Zhu *et al*. (2013)
**BRASSINOSTEROID**	Exogenous application of 24-epiBL	Enhanced the activity of antioxidant enzymes and ameliorated Ni-stress	*Brassica juncea*	Kanwar *et al*. (2013)
			*Raphanus sativus*	Sharma *et al*. (2011a)
			*Vigna radiate*	Yusuf *et al*. (2012)
	Exogenous application of 28-homoBL	Elevated CAT, POD, and SOD activity, protecting against Ni toxicity	Wheat	Yusuf *et al*. (2010)
	Exogenous application of homoBL	Improved Cd-tolerance by increasing activity of antioxidant enzymes (CAT, POD and SOD)	*Brassica juncea*	Hayat *et al*. (2007)
	Exogenous application of 24-epiBL	Enhanced Cd tolerance	*Phaseolus vulgaris*	Rady (2011)
	Exogenous application of 28-homoBL/ 24-epiBL	Reduced damage under Cd stress	Tomatoes	Hasan *et al*. (2011)
	Exogenous application of BRs	Enhanced antioxidant system activity and improved fruit yield and quality under Cd stress	*Solanum lycopersicum*	Hayat (2012)
	Exogenous application with 28-homoBL	Induced protection against Cd stress	*Cicer arietinum*	Hasan *et al*. (2008)
	BR treatment	Enhanced antioxidant activity under heavy metal stress	*Brassica juncea*	Fariduddin *et al*. (2009)
			*Raphanus sativus*	Kapoor *et al*. (2014)
			Radish	Ramakrishna and Rao (2013)
				Anuradha and Rao (2007)
				Choudhary *et al*. (2011)
			Tomato	Rady and Osman (2012)
			*Raphanus sativus* L.	Sharma *et al*. (2011b)
**ETHYLENE**	Cu	Induced the expression of the ACS genes	Potatoes	Schlagnhaufer *et al*. (1997)
	Cr	Increased expression of four ethylene biosynthesis-related genes (ACS1, ACS2, ACO4, and ACO5)	Rice	Steffens (2014) Trinh *et al*. (2014)
	Cd	Induced the biosynthesis of ACC and ethylene	*Arabidopsis thaliana*	Schellingen *et al*. (2014)
	Pb	Increased transcript levels of *EIN2*	Arabidopsis	Cao *et al*. (2009)
	Li	Ethylene insensitive *etr1-1* and *ein3-3* mutants were less sensitive to stress	Arabidopsis	Bueso *et al*. (2007)

Considering the importance of plant growth hormones (*e.g*., abscisic acid, auxins, brassinosteroids and *ethylene*) for alleviating heavy metal stress, further research is expected to contribute to a deeper knowledge on the *endogenous* regulation of plant hormone metabolism in such harmful conditions. There are several studies already in support of the importance of plant hormones under heavy metal exposure as well as other abiotic stresses. It is also interesting to note that the critical plant tolerance achieved by phytohormones can be promoted directly or indirectly. For example, ethylene signaling is mediated by EIN2, and some studies also show that the *EIN2* gene mediates Pb resistance through AtPDR12, an efflux pump at the plasma membrane. But phytohormone tolerance can also be induced indirectly, as the one promoted by brassinosteroids, which improves antioxidant systems efficiency in removing ROS and, as a consequence, attenuates detrimental effects from heavy metal stress. In the same way, hormonal treatments were not able to overcome the adverse effects of heavy metals on plant nutrient acquisition, but they were able to efficiently inhibit heavy metal incorporation, such as in stomatal closure guided by ABA that ultimately decrease toxic metals uptake and translocation from roots to shoots.

In summary, improving our knowledge of hormone metabolism in plants is critical for the development of new physiological, biochemical and biotechnological approaches towards mitigating the enormous spectra of abiotic stresses we see in today's environment, including heavy metal toxicity. With challenges such as climate change, which is expected to reduce crop yield in many areas, such information could prove critical for maintaining a reliable food supply for an ever-growing human population
